# Artificial Liver Support System Improves Short-Term Outcomes of Patients with HBV-Associated Acute-on-Chronic Liver Failure: A Propensity Score Analysis

**DOI:** 10.1155/2019/3757149

**Published:** 2019-11-29

**Authors:** Lan-Lan Xiao, Xiao-Wei Xu, Kai-Zhou Huang, Ya-Lei Zhao, Ling-Jian Zhang, Lan-Juan Li

**Affiliations:** State Key Laboratory for Diagnosis and Treatment of Infectious Diseases, Nationwide Clinical Research Center for Infectious Diseases, Collaborative Innovation Center for Diagnosis and Treatment of Infectious Diseases, The First Affiliated Hospital, College of Medicine, Zhejiang University, Hangzhou, China

## Abstract

**Background:**

Hepatitis B virus-associated acute-on-chronic liver failure (HBV-ALCF) is a complicated syndrome with extremely high short-term mortality. The artificial liver support system (ALSS) may improve the liver function for patients with HBV-ACLF, but the data on its short-term outcomes are insufficient in China.

**Methods:**

We recruited HBV-ACLF patients in this nationwide, multicenter, retrospective study. Patients with HBV-ACLF were diagnosed by the COSSH-ACLF criteria. Propensity score matching (PSM) analysis was used to generate compared pairs. The short-term (28/90 days) survival rates between the standard medical therapy (SMT) group and ALSS group were calculated using a Kaplan–Meier graph.

**Result:**

In total, 790 patients with HBV-ACLF were included in this retrospective study; 412 patients received SMT only (SMT group), and 378 patients received SMT and ALSS treatment (ALSS group). PSM generated 310 pairs and eliminated the baseline differences between the two groups (*p* > 0.05 for all baseline variables). The probabilities of survival on day 28 were 65.2% (205/310) in the ALSS group and 59.0% (185/310) in the SMT group; on day 90, they were 51.0% (163/310) and 42.3% (136/310). The short-term (28/90 days) survival rates of the ALSS group were significantly higher than those of the SMT group (*p*=0.0452 and *p*=0.0187, respectively). Compared to receiving SMT alone, treatment with ALSS was associated with a significant reduction in serum bilirubin levels and the model for end-stage liver disease (MELD) scores at day 7 and day 28. Multivariate logistic regression analysis revealed that older age, high total bilirubin (T-Bil), low albumin, high ALT, high MELD scores, and high COSSH-ACLF grade were independent baseline factors associated with poor prognosis.

**Conclusions:**

This retrospective study found that compared to SMT, the ALSS improved the short-term (28/90 days) survival rates and laboratory parameters in HBV-ACLF patients. The ALSS had a better therapeutic effect than SMT for patients with HBV-ACLF in China.

## 1. Introduction

Chronic hepatitis B (CHB) is a major public health challenge in China, with an estimated 78 million chronic carriers and 28 million patients with active hepatitis [1]. CHB is a significant risk factor that accounts for nearly 45% of cases of hepatocellular carcinoma (HCC) and 30% of cases of cirrhosis, causing nearly 1 million deaths each year worldwide [2, 3]. The high prevalence of CHB causes that hepatitis B virus (HBV) infection absolutely predominated in the etiologies of ACLF, accounting for 96.5% cases in China, while alcoholism is the most common etiologies of ACLF in western developed countries [4, 5].

HBV-related ACLF (HBV-ALCF) is a complicated syndrome with high short-term mortality (40–70% without liver transplantation) that develops in patients with HBV-related chronic liver disease [6, 7].

In past decades, a series of artificial liver support systems (ALSSs) have been applied in liver failure which aim to detoxify blood and compensate liver function for patients with liver failure. Molecular adsorbent recirculating system (MARS) [8, 9] and Prometheus [10, 11] are the most widely used ALSS. Li's artificial liver system (Li-ALS, the low-volume plasma exchange-centered ALSS) was designed by Professor Li's team since 1986 [12], which is mainly used in China. Some studies demonstrated that ALSSs could detoxify and ameliorate hepatic encephalopathy during acute liver failure (ALF) [11, 13, 14]. However, several large randomized trials noted that patients with ALF [15] or ACLF [16, 17], supported with ALSSs, did not show an increased short-term survival. A single-center study conducted in China reported that ALSS improved 90 days and 5 years outcomes of patients with HBV-ACLF [18].

In this study, we conducted a nationwide, multicenter, retrospective study to test whether ALSSs could improve the short-term (28/90 days) outcomes in patients with HBV-ALCF in China and identify predictive factors for the prognosis of these patients.

## 2. Methods

### 2.1. Patients

This is a retrospective cohort study where all data were fully anonymized before access. We recruited hospitalized patients with HBV-ACLF from 11 liver centers of Chinese University hospitals between January 2014 and May 2017. The following clinical data were collected: demographic data (age, sex, and body mass index), cirrhosis, laboratory measurements (HBV-DNA level, ALT, total bilirubin (T-Bil), INR, and creatinine), hepatic encephalopathy (HE), nucleos(t)ide analogs (NA), and survival time. The liver disease severity was assessed using the MELD scores and COSSH-ACLF grade (Chinese Group on the Study of Severe Hepatitis B-ACLF). The study was approved by the Ethics Committee of the First Affiliated Hospital, Zhejiang University School of Medicine.

### 2.2. Inclusion and Exclusion Criteria

The inclusion criteria were as follows:

Patients diagnosed with CHB [19] (HBV surface antigen-positive ≥6 months; serum HBV-DNA ≥20 000 IU/mL; a liver biopsy showing chronic hepatitis) and reached at least ACLF grade 1 diagnosed by the COSSH-ACLF criteria [6]:

1. ACLF grade 1: patients with kidney failure alone; patients with single liver failure (total bilirubin ≥12 mg/dL) with an international normalized ratio (INR) ≥1.5 and/or kidney dysfunction and/or HE grade I or II; patients with single type of organ failure of the coagulation, circulatory, or respiratory systems and/or kidney dysfunction and/or HE grade I or II; and patients with cerebral failure alone plus kidney dysfunction
2. ACLF grade 2: patients with failure of two organ systems
3. ACLF grade 3: patients with failure of 3 or more organ systems

The exclusion criteria were as follows:

1. Younger than 18 years or older than 80 years
2. Received a liver transplant
3. Human immunodeficiency virus infection
4. Diagnosed with HCC or another tumor
5. Dead within 3 days
6. Had a severe comorbidity that could affect survival

### 2.3. Therapies

According to the Diagnostic and Treatment Guideline for Liver Failure 2012 (Guideline (2012)) [20], the treatment for severe liver disease mainly consists of restoring and preserving vital organ function and slowing down the progression of multiple organ failure. The standard medical therapy (SMT) included a high-calorie diet; enteral nutrition is recommended; correction hypoproteinemia; correction water-electrolyte and acid-base balance; nucleoside analogs for HBV-DNA-positive patients; anti-infective therapy for infection; restricted protein diet; lactulose, ammonia drugs, and L-ornithine aspartate for HE; diuretics and tolvaptan for ascites; tubular active drugs; maintenance of arterial blood pressure and water restriction for hepatorenal syndrome; and oxygen therapy for hepatopulmonary syndrome.

According to the Guideline (2012), patients with early- or middle-stage liver failure were advised to receive ALSS treatment. For patients with early liver failure, PE was applied; for patients with metaphase hepatic failure, continuous blood purification (CBP) was applied; for patients with brain edema or renal failure or imbalance of water and electrolytes, CBP or plasma diafiltration (PDF) was applied; for patients with hyperbilirubinemia, plasma bilirubin absorption (PBA) was applied. The ALSS sessions were scheduled as follows: the ALSS was usually performed in 48 hours after diagnosis. ALSS treatment was performed daily on the first 2 or 3 days; future treatments were offered according to the patients' condition. Overall, 841 ALSS treatment sessions were applied in 310 patients (average of 2.7 sessions per patient, ranging from 1 to 8 sessions). 9 (2.9%) patients received PE (2–3 h/per session), and 301 (97.1%) patients received continuous renal replacement therapy (CRRT, 8 h/per session).

### 2.4. Statistical Analysis

Propensity score matching analysis was used to eliminate bias between the two groups. Propensity scores were computed using the following variables: age; sex; serum levels of HBV-DNA, ALT, T-Bil, and albumin; platelet count; white cell count; creatinine; INR; serum sodium; cirrhosis; HE; MELD score; and COSSH-ACLF grade. For propensity score matching, a nearest-neighbor 1 : 1 matching scheme was used. Categorical data were compared using the *χ*^2^ test or Fisher's exact test. Continuous variables were compared using the Mann–Whitney *U* test. The survival rate at 28 days and 90 days were calculated using a Kaplan–Meier graph. The difference in the survival rate was compared using a log-rank test. The relationship between baseline parameters and 28-day survival was studied using a multivariate logistic regression model. All statistical analyses were performed using IBM SPSS v. 24.0 for Windows (IBM Corp., Armonk, NY, USA). *p* values are two-tailed, and values less than 0.05 were considered statistically significant.

## 3. Results

### 3.1. Patient Characteristics

**Figure 1:**
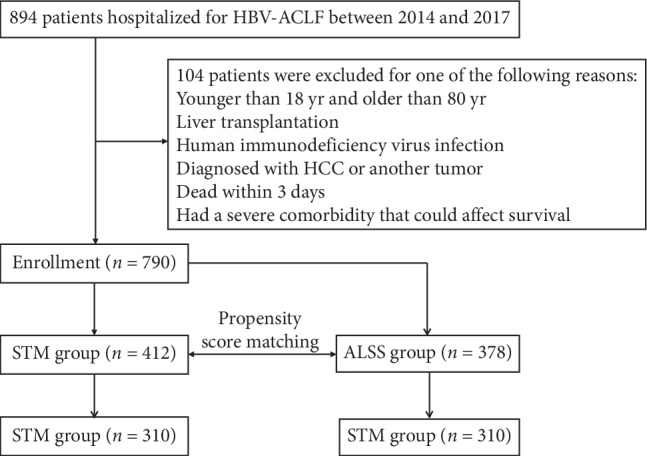
Flowchart of the patient selection process. HBV-ACLF, hepatitis B virus-related acute-on-chronic liver failure; LT, liver transplantation; STM, standard medical therapy; ALSS, artificial liver support system.

A total of 790 patients with HBV-ACLF were included in the study; 412 patients received standard treatment (SMT group) and 378 patients received ALSS treatment (ALSS group, Figure 1). Among them, 598 (85.3%) patients were male and 369 (52.5%) patients had no cirrhosis. Before matching, the baseline characteristics between two groups that differed significantly, such as ALT, T-Bil, and platelet count. Propensity score matching analysis generated 173 pairs, and the baseline characteristics of the pairs were balanced, with *p* > 0.05 for all baseline variables. After matching, the mean (SD) age of the patients in the SMT and ALSS groups was 45.4 (11.1) and 46.8 (11.3) for male, and the mean (SD) MELD scores were 25.1 (5.2) and 24.9 (5.6). In the SMT group, 118 (38.1%) patients had cirrhosis, and in the ALSS group, 133 (42.9%) patients had cirrhosis. All patients received NAs after diagnosis. In the SMT group, 155 patients received 100 mg of lamivudine (LAM) daily and 155 patients received 0.5 mg of entecavir (ETV) daily. In the ALSS group, 131 patients received 100 mg of LAM daily; 157 patients received 0.5 mg of ETV daily; 14 patients received 300 mg of tenofovir daily; and 8 patients received 600 mg of telbivudine daily. The characteristics of all patients are shown in Table 1.

### 3.2. Survival Rates

**Figure 2:**
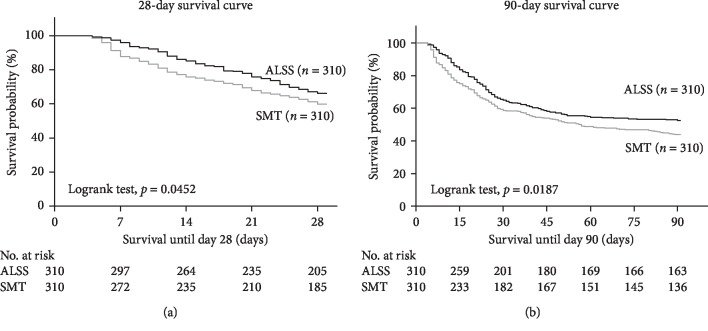
Short-term (28/90 days) survival curves for patients with HBV-ACLF. (a) 28-day survival curves for patients in the SMT and ALSS groups. (b) 90-day survival curves for patients in the SMT and ALSS groups. HBV-ACLF, hepatitis B virus-related acute-on-chronic liver failure; STM, standard medical therapy; ALSS, artificial liver support system.

For all patients, the short-term (28/90 days) survival rates were 60.0% (474/790) and 45.8% (362/790). The 28-day survival rates were 65.2% (205/310) in the ALSS group and 59.0% (185/310) in the SMT group; the 90-day survival rates were 51.0% (163/310) and 42.3% (136/310), respectively. The short-term (28/90 days) mortality rate was significantly lower in the ALSS group than in the SMT group (*p*=0.0452; *p*=0.0187, Figures 2(a) and 2(b)).

In the SMT group, patients treated with entecavir had similar short-term survival rates than patients treated with lamivudine (*p* > 0.05). In the ALSS group, patients treated with entecavir had higher short-term (28/90 days) survival rates than patients treated with lamivudine (77.1%: 64.1%; 62.4%: 49.6%, *p* < 0.05, Table 2). The short-term (28/90 days) survival rates in the patients with noncirrhotic HBV-ACLF (63.6%/49.65%) and cirrhotic HBV-ACLF (61.8%/46.2%) were not significantly different.

The 28-day survival rates of patients with ACLF grades 1–3 were 72.5%, 37.2%, and 0 in the SMT group and 78.5%, 43.5%, and 11.1% in the ALSS group. The 90-day survival rates of patients with ACLF grades 1–3 were 55.6%, 22.3%, and 0 in the SMT group and 65.1%, 29.3%, and 0% in the ALSS group (Table 3).

### 3.3. Changes in Laboratory Parameters at Day 7 and Day 28

The effect of treatment on laboratory parameters at day 7 and day 28 is shown in Tables 4 and 5, respectively. Compared with the SMT treated alone group, treatment with ALSS was associated with a significant reduction in serum T-Bil levels and MELD scores at both day 7 and day 28; however, the serum creatinine only decreased at day 7. The remaining analyzed parameters showed no significant difference between the two groups.

### 3.4. Risk Factors on 28-Day Survival

Using multivariate logistic regression analysis, the independent baseline risk factors for 28-day survival were identified as age, T-Bil, low albumin, ALT, MELD score, and COSSH-ACLF grade (Table 6). Cirrhosis, INR, serum creatinine, and platelet count were not independent predictors of 28-day survival.

## 4. Discussion

HBV-ACLF is observed in populations with HBV-related chronic liver disease. Liver transplantation is the most effective therapy for patients with liver failure; however, less than 30% of patients have access to transplantation because of donor shortages and the extremely poor prognosis of HBV-ACLF [21]. Although NA treatment could effectively decrease the 3-month mortality for patients with HBV-ACLF, NAs are only valid in patients with MELD scores less than 30 [22, 23]. In addition, mutations resistant to NAs are frequent precipitating events of HBV-ACLF, which is related to high mortality [24]. The development of ACLF includes the accumulation of various metabolites and toxins that vary in size, distribution volume, lipophilicity, and protein-binding abilities [25, 26]. Most ALSSs are capable of correcting the hemato-microenvironment, such as detoxification, synthesis, immune regulation, and reducing mortality in patients with ACLF, when compared with SMT [27]. Therefore, ALSS has been recommended as an important method to treat ACLF.

HBV-ACLF is a special type of ACLF and is the most frequent ACLF in China. This large, multicenter, nationwide, historical retrospective study showed that treatment with ALSS in patients with HBV-ACLF remarkably improved their short-term (28/90 days) survival rates compared with those receiving SMT only. The results indicated that ALSS is effective at removing the toxic substances from plasma that accumulate in patients with HBV-ACLF (confirmed by the significant decreased in T-Bil), correcting coagulopathy (confirmed by the INR decline), and alleviating renal failure (confirmed by the creatinine decline). The functions of synthesis and immune regulation have been well established in pigs with ALF, although further clinical studies are needed.

The ALSS performed in this study innovatively used plasma separators with an aperture of about 1/10 (membrane pore size = 0.03 *μ*m) of that of a normal plasma separator for direct PE, which could remove toxic substances effectively for patients with liver failure, retain important plasma components, and reduce the plasma dosage [28]. Although high-volume plasma (HVP, approximately 8 L) exchange has a beneficial therapeutic effect on patients with ALF [26, 29], it is usually performed over more than two sessions for patients with ACLF, which results in a shortage of fresh plasma. Moreover, the HVP removes almost all elements of plasma; part of them are beneficial substances (such as hepatic growth factor) for liver regeneration [30]. In this study, the ALSS using lower-volume plasma (LVP) exchange in which the total volume of exchanged fresh plasma is approximately 1500 mL [12]. The LVP is usually the first step of the ALSS, and subsequent adsorption and hemofiltration circulation are conducted through autologous plasma derived from waste plasma. The waste plasma is purified, which avoids wasting massive plasma and reduces the loss of essential substances. However, whether low-volume PE provides better results compared with high-volume PE remains unclear.

Selection of suitable inclusion criteria is crucial to evaluate the efficacy of ALSS for patients with HBV-ACLF. HBV-ACLF is an extremely special type of ACLF with some distinctive characteristics. Patients with HBV-ACLF have poorer prognosis than patients with non-HBV-ACLF (the 28/90 days mortality rates were 60.2% vs 52.1% and 73.9% vs 69.7%) [6]. Patients with HBV-ACLF have a higher incidence of liver and coagulation failure [31, 32], whereas kidney failure and cerebral failure are the most common types of organ failure in patients with non-HBV-ACLF. Reactivation of HBV, an acute hepatic insult, is the leading cause of HBV-ACLF in the Asian region [33–36]. The European Association for the Study of the Liver (EASL) criteria and the American Association for the Study of Liver Diseases (AASLD) criteria are the major criteria for ACLF in patients from Europe and North America, where alcoholic liver disease is the major etiology [7, 37]. These criteria may not apply to China, where hepatitis B virus infection is the major etiology in China. The COSSH criteria were established based on a large Chinese HBV-ACLF group which is in accordance with our study group. Therefore, we chose the COSSH criteria as the inclusion criteria.

A previous study showed that the first leading cause of HBV-ACLF was spontaneous severe acute exacerbation of CHB (62.5%) and the second leading cause was alcohol (15.4%) in Asia [5]. The oral NA therapy effectively suppresses viral DNA and prevents the progression of liver inflammation; therefore, rapid initiation of oral NA treatment in patients with HBV-ACLF is recommended widely [38, 39]. LAM and ETV are both widely used in China, though LAM has less potency and has higher resistance (LAM: 70%; ETV: 1.2%) than ETV [39].

This study revealed that compared with LAM, ETV did not improve the short-term survival rates in HBV-ACLF, neither in the SMT group nor ALSS group. These results were similar to a largest meta-analysis, which demonstrated a comparable short-term mortality (within 4 months) of LAM and ETV; however, ETV revealed a more favorable long-term (beyond 4 months) outcome than LAM in patients with HBV-ACLF [40]. The results of multivariate logistic regression showed that cirrhosis is not a risk-independent predictor of 28-day survival. We also found a similarly short-term survival rate in patients with both cirrhotic HBV-ACLF (61.8%/46.2%) and noncirrhotic HBV-ACLF (63.6%/49.65%). However, this result was not in line with that of Wu's study, which indicated that cirrhosis patients had superior 28-day survival rate than noncirrhosis patients (47.9%: 39.8%, *p* < 0.05) [6]. The contradictory outcomes might result from small population samples of this study. In previous studies, T-Bil and platelet levels were found as independent risk factors of mortality among HBV-ACLF patients; however, in our study, the significant difference was only found in single-factor logistic regression analysis.

In conclusion, among patients with HBV-ACLF in China, ALSS has better therapeutic effect than SMT. Though the treatment for HBV-ACLF haas improved over the past three decades the short-term mortality of ACLF remains high. Therefore, more effective therapeutic methods should be investigated.

## Figures and Tables

**Table 1:** Clinical characteristics of the two groups studied at baseline.

Characteristics	Entire cohort			Propensity score-matched cohort		
	SMT (*n* = 412)	ALSS (*n* = 378)	*p* value	SMT (*n* = 310)	ALSS (*n* = 310)	*p* value
Age (y)	44.0 (10.6)	46.9 (11.4)	0.097	45.4 (11.1)	46.8 (11.3)	0.286
Sex, male	351 (85.2)	337 (86.0)	0.097	271 (87.4)	274 (84.6)	0.712
HBV-DNA (log copies/mL)	5.1 (1.6)	5.1 (2.0)	0.641	5.2 (1.1)	5.1 (1.9)	0.705
Alanine transaminase level (IU/L)	388.5 (277.4)	341.1 (326.5)	0.010	376.3 (239.8)	360.4 (300.1)	0.695
Total bilirubin level (mg/dl)	22.1 (7.4)	24.4 (7.0)	0.002	22.7 (7.4)	23.9 (7.0)	0.121
Serum albumin level (g/L)	33.1 (5.9)	32.1 (6.3)	0.027	33.1 (5.6)	31.9 (5.4)	0.149
Platelet count (×105/mm^3^) (SD)	107.3 (56.3)	103.8 (48.1)	0.015	105.3 (57.9)	104.7 (45.8)	0.893
White cell count (×109/L)	9.1 (21.3)	7.9 (3.3)	0.018	9.4 (24.1)	7.6 (4.0)	0.197
Serum creatinine level (mg/dl)	82.3 (44.3)	76.5 (30.6)	0.006	72.6 (23.7)	68.5 (25.5)	0.088
International normalized ratio	2.5 (0.9)	2.5 (0.6)	0.759	2.3 (0.8)	2.4 (0.9)	0.687
Serum sodium (mEq/L)	137.6 (4.5)	136.0 (4.1)	<0.001	135.7 (4.9)	136.4 (3.7)	0.181
Hepatic encephalopathy ≥ grade II	9.5% (39/412)	13.8% (52/378)	0.059	10.3% (32/310)	14.2% (44/378)	0.142
Cirrhosis	158 (38.3)	161 (42.6)	0.225	118 (38.1)	133 (42.9)	0.220
MELD score	25.9 (5.2)	24.8 (5.6)	0.721	25.1 (5.2)	24.9 (5.6)	0.594
COSSH-ACLF grade			0.091			0.985
ACLF grade 1	226 (54.9)	235 (62.2)		207 (66.8)	209 (67.4)	
ACLF grade 2	174 (42.2)	131 (34.7)		94 (30.3)	92 (29.7)	
ACLF grade 3	12 (2.9)	12 (3.2)		9 (2.9)	9 (2.9)	
Nucleos(t)ide analogues			<0.001			<0.001
Lamivudine	205	167		155	131	
Entecavir	207	182		155	157	
Tenofovir	0	18		0	14	
Telbivudine	0	11		0	8	

Values are expressed as number and percentage or mean ± SD unless otherwise specified. SMT, standard medical therapy; ALSS, artificial liver support system; MELD, model for end-stage liver disease; COSSH-ACLF, Chinese Group on the Study of Severe Hepatitis B-ACLF.

**Table 2:** Short-term (28/90 days) survival rates for patients treated with lamivudine vs entecavir or patients with noncirrhotic HBV-ACLF vs cirrhotic HBV-ACLF.

Nucleos(t)ide analogues	28-day survival rate			90-day survival rate		
	SMT	ALSS	Overall	SMT	ALSS	Overall
Lamivudine	61.3% (95/155)	64.1% (84/131)	62.6% (179/286)	41.9% (65/155)	49.6% (65/131)	45.5 (130/286)
Entecavir	58.1% (90/155)	77.1% (121/157)	67.6% (211/312)	45.8% (71/155)	62.4% (98/157)	54.2% (169/312)
*p* value	0.563	0.016	0.794	0.492	0.029	0.033
Noncirrhotic HBV-ACLF	60.4% (116/192)	67.0% (120/179)	63.6% (236/371)	45.3% (87/192)	54.2% (97/179)	49.6% (184/371)
Cirrhotic HBV-ACLF	58.5% (69/118)	64.9% (85/131)	61.8% (154/249)	41.5% (49/118)	50.4% (66/131)	46.2% (115/249)
*p* value	0.735	0.692	0.656	0.514	0.507	0.405

**Table 3:** Short-term survival rates for patients in different COSSH-ACLF grades.

COSSH-ACLF	Prevalence	28-day survival rate	90-day survival rate
Total (*n* = 790)		60.0% (474/790)	45.8% (362/790)
ACLF grade 1	58.4% (461/790)	76.1% (351/461)	61.2% (282/461)
ACLF grade 2	38.6% (305/790)	40.0% (122/305)	26.2% (80/305)
ACLF grade 3	3.0% (24/790)	4.2% (1/24)	0 (0/24)
SMT (*n* = 310)		59.0% (185/310)	42.3% (136/310)
ACLF grade 1	66.8% (207/310)	72.5% (150/207)	55.6% (115/207)
ACLF grade 2	30.3% (94/310)	37.2% (35/94)	22.3% (21/94)
ACLF grade 3	2.9% (9/310)	0 (0/9)	0 (0/9)
ALSS (*n* = 310)		65.2% (205/310)	51.0% (163/310)
ACLF grade 1	67.4% (209/310)	78.5% (164/209)	65.1% (136/209)
ACLF grade 2	29.7% (92/310)	43.5% (40/92)	29.3% (27/92)
ACLF grade 3	2.9% (9/310)	11.1% (1/9)	0 (0/9)

SMT, standard medical therapy; ALSS, artificial liver support system.

**Table 4:** Effects of treatment on laboratory parameters at day 7.

Parameter	SMT (*n* = 272)	ALSS (*n* = 392)	*p* value
Serum bilirubin (mg/dl)			
Baseline	22.8 (7.3)	23.6 (7.0)	
Day 7	23.9 (7.5)	22.2 (6.8)	
Change from baseline	1.1 (7.2)	−1.4 (4.7)	0.008
Serum albumin (g/L)			
Baseline	33.6 (5.4)	32.1 (5.3)	
Day 7	33.8 (7.7)	34.1 (7.3)	
Change from baseline	0.2 (4.5)	2.0 (17.3)	0.067
Baseline international normalized ratio			
Baseline	2.3 (0.8)	2.4 (0.9)	
Day 7	2.2 (0.8)	2.2 (0.8)	
Change from baseline	−0.1 (0.50)	−0.2 (0.92)	0.064
Serum creatinine (*μ*mol/L)			
Baseline	72.6 (23.7)	68.5 (25.5)	
Day 7	75.9 (28.5)	66.2 (24.8)	
Change from baseline	3.3 (25.2)	−2.3 (17.9)	0.043
Alanine transaminase level (IU/L)			
Baseline	371.8 (237.1)	360.4 (300.1)	
Day 7	138.4 (137.1)	159.7 (196.7)	
Change from baseline	−233.4 (293.7)	−200.7 (241.8)	0.071
MELD score			
Baseline	25.2 (5.1)	25.0 (5.6)	
Day 7	25.1 (4.7)	24.3 (5.8)	
Change from baseline	−0.1 (3.4)	−0.7 (6.2)	0.036

SMT, standard medical therapy; ALSS, artificial liver support system; MLED, model for end-stage liver disease.

**Table 5:** Effects of treatment on laboratory parameters at day 28.

Parameter	SMT (*n* = 185)	ALSS (*n* = 205)	*p* value
Serum bilirubin (mg/dl)			
Baseline	22.1 (7.1)	23.2 (6.8)	<0.001
Day 28	15.2 (10.1)	22.8 (10.8)	
Change from baseline	−4.4 (9.5)	−6.9 (9.5)	
Serum albumin (g/L)			
Baseline	33.6 (5.4)	32.1 (5.3)	
Day 28	33.8 (7.7)	34.1 (7.3)	
Change from baseline	0.2 (4.5)	2.0 (17.3)	0.067
Baseline international normalized ratio			
Baseline	2.3 (0.6)	2.3 (0.6)	
Day 28	1.7 (0.7)	1.7 (0.7)	
Change from baseline	−0.5 (0.7)	−0.5 (0.7)	0.975
Serum creatinine (*μ*mol/L)			
Baseline	73.2 (22.7)	70.9 (22.8)	
Day 28	72.4 (34.5)	70.8 (25.9)	
Change from baseline	−0.8 (34.8)	−0.1 (23.4)	0.088
ALT (IU/L)			
Baseline	356.2 (242.5)	367.3 (287.4)	
Day 7	60.8 (89.7)	44.9 (26.7)	
Change from baseline	−295.4 (293.7)	−322.4 (241.8)	0.085
MELD score			
Baseline	25.2 (4.2)	25.4 (4.8)	
Day 28	17.7 (9.5)	16.8 (7.8)	
Change from baseline	−7.5 (8.2)	−8.6 (6.4)	0.042

SMT, standard medical therapy; ALSS, artificial liver support system; MLED, model for end-stage liver disease.

**Table 6:** Multivariate logistic regression model investigating independent risk factors on 28-day survival.

Variable	Reference	OR (CI 95 %)	*p* value
Age	Per year	1.017 (1.002–1.033)	0.022
Total bilirubin	Per unit increase	1.062 (1.035–1.088)	<0.001
Alanine transaminase	Per unit increase	1.001 (1.000–1.0001)	0.001
Albumin	Per unit decline	1.053 (1.011–1.074)	0.009
MELD score	Per point increase	1.059 (1.011–1.109)	0.015
COSSH-ACLF grade	Per rank increase	2.683 (1.792–4.017)	<0.001

MLED, model for end-stage liver disease; COSSH-ACLF, Chinese Group on the Study of Severe Hepatitis B-ACLF.

## Data Availability

All relevant data are within the paper and its supporting information file.
