# Corrigendum to “Artificial Liver Support System Improves Short-Term Outcomes of Patients with HBV-Associated Acute-on-Chronic Liver Failure: A Propensity Score Analysis”

**DOI:** 10.1155/2021/9875940

**Published:** 2021-06-08

**Authors:** Lan-Lan Xiao, Xiao-Wei Xu, Kai-Zhou Huang, Ya-Lei Zhao, Ling-Jian Zhang, Lan-Juan Li

**Affiliations:** State Key Laboratory for Diagnosis and Treatment of Infectious Diseases, Nationwide Clinical Research Center for Infectious Diseases, Collaborative Innovation Center for Diagnosis and Treatment of Infectious Diseases, The First Affiliated Hospital, College of Medicine, Zhejiang University, Hangzhou, China

In the article titled “Artificial Liver Support System Improves Short-Term Outcomes of Patients with HBV-Associated Acute-on-Chronic Liver Failure: A Propensity Score Analysis” [1], some information was omitted in the Acknowledgments section, which should be corrected as follows:

The authors would like to thank Jian-Rong Huang, Shao-Jie Xin, Zhong-Ping Duan, Yu-Ming Wang, Qing Xie, Zhi-Liang Gao, Jian-He Gan, Tao Han, Shu-Mei Lin, Chen Pan, Li Zhuang, and Yong-Ping Chen for patient data collection. This work was supported by the Science & Technology Key Program of Zhejiang China (#2017C03051).
